# Linear-*g*-hyperbranched and cyclodextrin-based amphiphilic block copolymer as a multifunctional nanocarrier

**DOI:** 10.3762/bjoc.10.284

**Published:** 2014-11-18

**Authors:** Yamei Zhao, Wei Tian, Guang Yang, Xiaodong Fan

**Affiliations:** 1 College of Environmental and Chemical Engineering, Xi’an Polytechnic University, Xi’an, 710048, P. R. China,; 2 The Key Laboratory of Space Applied Physics and Chemistry, Ministry of Education and Shanxi Key Laboratory of Macromolecular Science and Technology, School of Science, Northwestern Polytechnical University, Xi’an, 710072, P. R. China

**Keywords:** amphiphilic block copolymer, cyclodextrin polymer, hyperbranched polymer, multifunctionality, polymer nanocarrier

## Abstract

In this paper, a novel, multifunctional polymer nanocarrier was designed to provide adequate volume for high drug loading, to afford a multiregion encapsulation ability, and to achieve controlled drug release. An amphiphilic, triblock polymer (ABC) with hyperbranched polycarbonsilane (HBPCSi) and β-cyclodextrin (β-CD) moieties were first synthesized by the combination of a two-step reversible addition-fragmentation transfer polymerization into a pseudo-one-step hydrosilylation and quaternization reaction. The ABC then self-assembled into stable micelles with a core–shell structure in aqueous solution. These resulting micelles are multifunctional nanocarriers which possess higher drug loading capability due to the introduction of HBPCSi segments and β-CD moieties, and exhibit controlled drug release based on the diffusion release mechanism. The novel multifunctional nanocarrier may be applicable to produce highly efficient and specialized delivery systems for drugs, genes, and diagnostic agents.

## Introduction

In recent years, many nanostructured materials (such as polymeric micelles) used as pharmaceutical nanocarriers have been applied in different fields of biomedicine for controlled release, diagnostics, imaging, treatment and prophylactics [1–5]. Although nanocarriers indeed offer several advantages and demonstrate a broad variety of useful properties compared to conventional chemotherapeutic agents [6–7], unresolved issues still exist in the drug delivery system (DDS), such as the conflict between circulation times and accumulation of drug in the target site [8]. For this reason, the nanocarriers are required to combine various properties or functions in one system.

Recently, the concept of multifunctional nanocarriers that can simultaneously perform multiple functions, such as longevity, targeting, stimuli sensitivity, and contrast properties, raising increasing attention [3,6–7,9–14]. Compared with other multifunctional nanocarriers reported in the literature, polymeric micelles formed by amphiphilic block copolymers (ABC) may possess many advantages including water-solubility, nontoxicity, selectivity of targeting, prolonged blood circulation time, etc. Therefore, they have attracted much attention [6,15–19]. However, to develop an efficient DDS constructed from ABC micelles, it is essential to consider some important factors, such as high drug loading and controlled release. Many of the reported ABC systems cannot provide enough volume for high drug loading, and as a result, a large amount of a nanocarrier is required to administer a desired drug dose [31]. Furthermore, sustained release, which is needed to control the release of drugs to the desired site within a given period of time, is very important and necessary for DDS [20]. On the other hand, some new functionalities for ABC micelles could be developed and expanded. For example, multiregion encapsulation ability, where various drugs can be encapsulated simultaneously in the core and shell layers of ABC micelles, could be an advantageous property. This property may be beneficial for further enhancement of the drug loading capability of the nanocarrier. Therefore, it is still very challenging and highly desirable to develop an ABC system to overcome these above-mentioned problems and expand new functionalities.

**Scheme 1:**
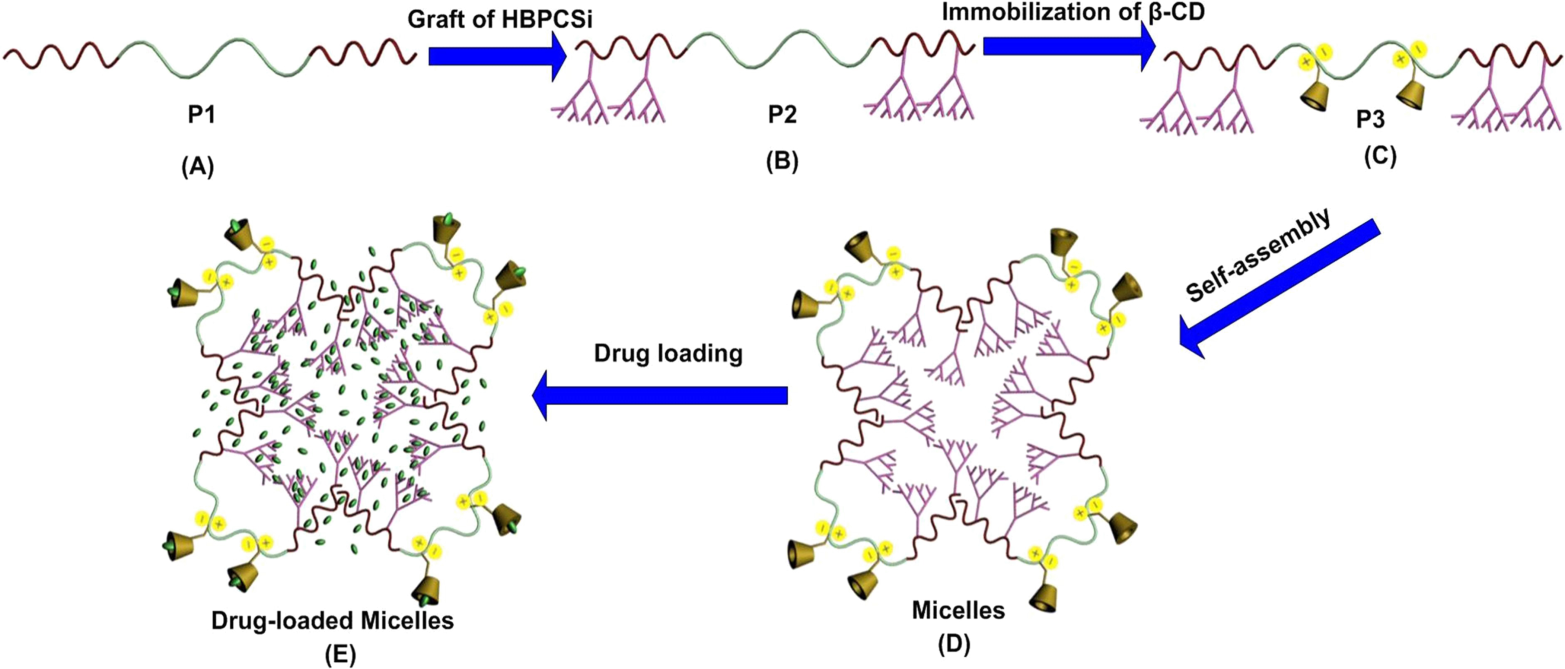
Schematic representation of the details for the construction of a multifunctional nanocarrier used for drug loading.

The goal of this research is to engineer a multifunctional nanocarrier for addressing the above issues simultaneously. Specifically, a novel ABC based on the linear-hyperbranched-cyclodextrin (CD) ternary system (Scheme 1A–C) was designed, which can self-assemble into the core–shell structured micelles (Scheme 1D) and simultaneously perform three functionalities including high loading, multiregion encapsulation, and controlled release (Scheme 1E). As prepared ABC, consisting of two hydrophobic poly[2-hydroxyethyl methacrylate] (PHEMA) blocks and one hydrophilic poly(*N*,*N*-dimethylaminoethyl methacrylate) (PDMAEMA) block (Scheme 1A, P1), allows for the self-assembly and controlled release by taking advantage of the amphiphilic structure and the stimuli-responsive property of PDMAEMA block, respectively. When the hyperbranched polycarbonsilane (HBPCSi) is grafted onto the side chain of PHEMA blocks (Scheme 1B, P2), the drug loading capacity of the polymer nanocarrier may be enhanced due to the existence of inner cavities from the hyperbranched topological structure. Additionally, the outer shell functionality of the polymer nanocarrier can be expanded to encapsulate more drugs due to the introduction of the β-CD moieties (Scheme 1C, P3).

Based on the above considerations, P1 was first synthesized via reversible addition–fragmentation transfer polymerization (RAFT), where HEMA and DMAEMA were used as the first and second monomer, respectively. To obtain P2, HBPCSi was then grafted onto the side chain of two PHEMA blocks via a pseudo-one-step hydrosilylation reaction. Finally, modified β-CD monomers were bonded to the amino groups of PDMAEMA block side chain by the action of ionic bonds (P3). The P3 self-assembled into stable micelles with a core–shell structure in aqueous solution. The resulting multifunctional, polymeric micelle nanocarriers provide higher drug loading capacity compared to their counterparts due to the introduction of the HBPCSi segments. Furthermore, drug molecules can be simultaneously encapsulated into the hydrophobic core layer and the hydrophilic shell layer of micelles by taking advantage of the β-CD cavities. Additionally, longevity and controlled drug release abilities are possible as a result of the PDMAEMA block.

## Results and Discussion

### Synthesis of ABC with HBPCSi and β-CD (P3)

P1 was first synthesized via a two-step RAFT polymerization and the subsequent acylation reaction (Scheme S1). As illustrated in Supporting Information File 1, Scheme S1, the PHEMA-based macro chain transfer agent (PHEMA-macroCTA) carrying abundant hydroxy groups was prepared by using *S*,*S*’-bis(α,α’-dimethylacetic acid) trithiocarbonate (BDATTC) as a highly efficient chain transfer agent [21]. Subsequently, the triblock copolymer (denoted as PHEMA-*b*-PDMAEMA-*b*-PHEMA) was easily obtained by using PHEMA-macroCTA. Finally, P1 was synthesized by the reaction of acryloyl chloride with the pendent hydroxy groups of PHEMA blocks. The introduction of double bonds further provides the possibility for grafting a reaction. Their macromolecular structures were well-defined as confirmed by ^1^H NMR, ^13^C NMR and FTIR (Supporting Information File 1, Figures S1–S3). The average degree of polymerization (DP) of PHEMA and PDMAEMA blocks was calculated from size exclusion chromatography/multiangle laser light scattering (SEC/MALLS) results (Supporting Information File 1, Table S1 and Figure S4). Obviously, the ratio of chain length of PDMAEMA to PHEMA, 5:1, was high. The synthesis of P2 was then obtained via a pseudo-one-step hydrosilylation reaction (Supporting Information File 1, Scheme S2), where the methyldiallylsilane was used as an AB_2_ monomer, and the two, divided PHEMA blocks containing the double bond groups were similar to the multifunctional cores. The DP of HBPCSi was approximately 430 as estimated by SEC/MALLS analysis (Supporting Information File 1, Table S1 and Figure S5), further indicating that the pseudo-one-step method was effective in this reaction. Although the structure of P2 is relatively complicated, ^1^H nuclear magnetic resonance (NMR), ^13^C NMR, and Fourier transform infrared spectroscopy (FTIR) spectra can still be used to confirm the existence of characteristic groups of HBPCSi (Supporting Information File 1, Figures S6 and S7).

Next, P3 was synthesized by the quaternization reaction of the amino groups of the PDMAEMA block side chain with monoiodide-substituted β-CD (mono-6-I-β-CD) (Supporting Information File 1, Scheme S3). The corresponding reaction mechanism was previously discussed [22]. ^1^H NMR, ^13^C NMR, and FTIR were employed to characterize the structure of P3 (Supporting Information File 1, Figures S8 and S9). In the ^1^H NMR spectrum of P3 (Supporting Information File 1, Figure S8a), new chemical shifts were found at δ 5.01 and δ 3.25–3.61 (protons from β-CD), indicating that the quaternization reaction was successfully conducted. Furthermore, the ^13^C NMR spectrum (Figure S8b) also confirmed the presence of the 6-ammonium-β-CD derivative in the polymer structure of P3. Compared with Supporting Information File 1, Figure S6b, the carbon atom peaks at δ 102.8, δ 82.9, δ 72.5–74.2, δ 71.1 and δ 60.8 can be assigned to C-1, C-4, C-3, C-5, C-2, C-6’ and C-6 in β-CD units (Supporting Information File 1, Figure S8b), respectively. Although P3 was successfully synthesized, the number of β-CD moieties conjugated into the PDMAEMA block was limited to 40 per P3 macromolecule, as determined by the SEC/MALLS data (Supporting Information File 1, Table S1 and Figure S10). This implies that only 40 repeating units of PDMAEMA segments were bonded by β-CD moieties, however, there are 1670 repeating units in total, indicating a relatively low supporting amount (about 2.4 wt %). This result may be attributed to the steric hindrance effect of the PDMAEMA block and mono-I-β-CD. However, if the pendent β-CD moieties are kept far apart from one another on the side chain of the PDMAEMA block, it should be much easier to complete the conformational transition in the micellization process.

### Self-assembly behaviour of P3 in aqueous solution

After the synthesis of P3 with amphiphilic characteristics, the corresponding micelle system with a core–shell structure can be formed in aqueous solution by self-assembly. This is driven by the strong hydrophobic–hydrophilic interactions between the inner core and the outer shell. The micelles were prepared by directly adding water to a DMF solution of P3 until the required amount of water for micelle formation was reached. Dynamic light scattering (DLS) and transmission electron microscopy (TEM) measurements were employed to obtain a deeper insight into the self-assembly morphology and size of P3 in aqueous solution.

**Figure 1:**
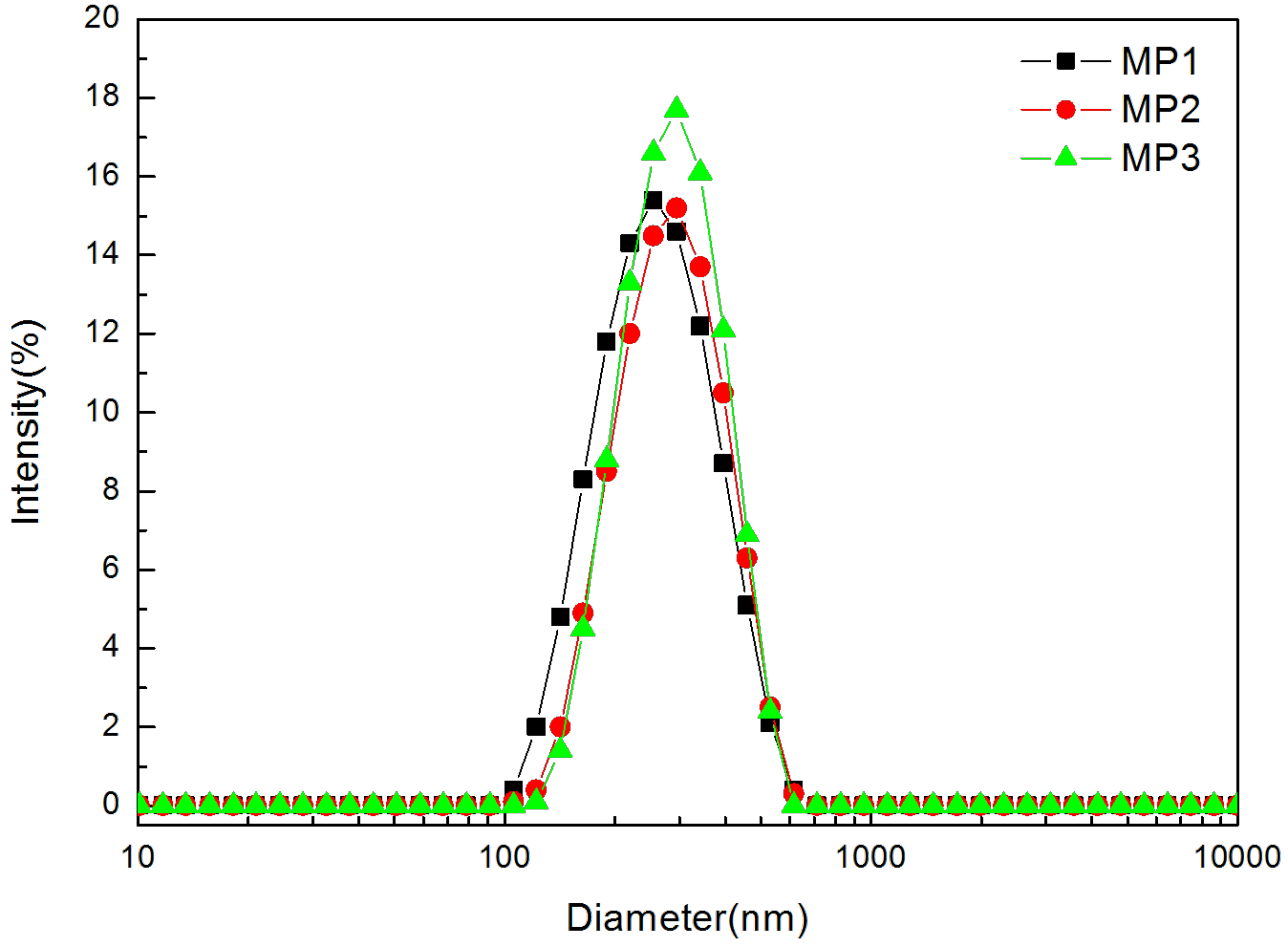
Particle size distributions of MP1, MP2, and MP3 at 25 °C as determined by DLS.

Figure 1 presents the diameter distributions of P1, P2 and P3 micelles (denoted as MP1, MP2, and MP3) in aqueous solution at 25 ºC. Compared with the Z–average diameter values of P1 (242 nm) and P2 (268 nm), P3 increased to 308 nm. This may be attributed to the attachment of the HBPCSi segments and β-CD moieties in the polymer structure of P3. Their PDI values confirmed a uniform micelle system (Table 1). The core–shell structures of these micelles were further confirmed by ζ-potential measurements. As seen from Table 1, MP1, MP2, and MP3 show positive values ranging from 24.3 to 15.0 mV at 25 ºC. This indicates that positive charges were located on the surfaces of these micelles. Generally, the presence of surface charges on micelles can result in a higher absolute value of the ζ-potential [23–24]. Thus, the positive charge of the current polymers should be ascribed to the presence of the PDMAEMA segments carrying tertiary amino groups. Therefore, when P1, P2 and P3 self-assembled into the micelles in aqueous solution, the PDMAEMA segments formed the outer shell of micelles, leading to the positive ζ-potential values. In addition, the ζ-potential value of the P3 solution was lower than those of P1 and P2. This may be attributed to the introduction of the mono-6-ammonium-β-CD derivative where the negative charges offset the partial positive charges of micelle surface. From this result, it can also be concluded that β-CD moieties were located on the outer shell of micelles.

**Table 1:** Physicochemical characteristics of MP1, MP2, and MP3 and LND-loaded micelles at 25 ºC in aqueous solution.

Sample index	D_z_^a^, nm	PDI^b^	ζ-potential^c^, mV

MP1	242	0.101	24.3
MP2	268	0.360	23.8
MP3	308	0.351	15.0
DLMP1	144	0.038	–
DLMP2	241	0.272	–
DLMP3	262	0.343	–

^a^The Z-average diameter, determined by DLS. ^b^The polydispersity index, determined by DLS. ^c^Determined by DLS.

**Figure 2:**
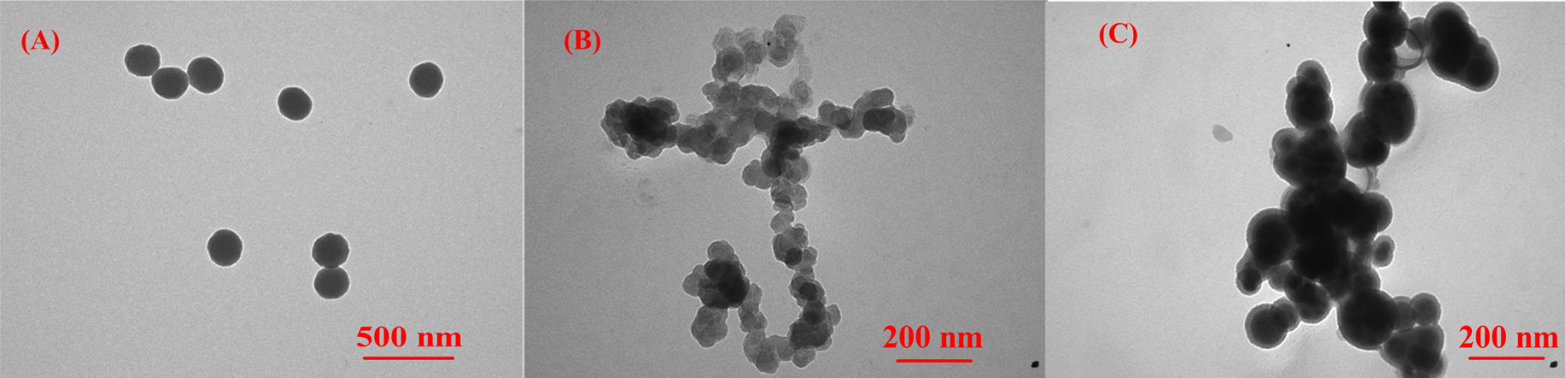
TEM images of P1 (A), P2 (B) and P3 (C) micelles.

To visually examine the formation of the micelles, TEM images of MP1, MP2, and MP3 are shown in Figure 2. Spherical micelles were observed for MP1 (Figure 2A), whereas the morphologies of MP2 and MP3 were not very regular (Figure 2B and C). Furthermore, as seen in Figure 2B and C, larger aggregates were formed due to the attachment of the HBPCSi segments and β-CD moieties. Large second aggregates from hyperbranched, polymer-based copolymers were also observed by Hong et al. [25]. However, the irregular morphologies and larger aggregates have no evident effect on the multifunctionality of MP2 and MP3 as nanocarriers, as will be confirmed in the next section.

### Multifunctional properties of nanocarriers

In order to address the high drug loading, multiregion encapsulation and controlled release capabilities of MP1–MP3, lonidamine (LND) as a hydrophobic anticancer drug was selected (Supporting Information File 1, Scheme S4). The encapsulation efficiency (EE) and loading content (LC) of LND-loaded micelles (DLMP1, DLMP2, and DLMP3) are presented in Table 2. It was found that the EE and LC of DLMP3 were significantly higher than those of DLMP1 and DLMP2. EE values were 88.9% vs 20.5% and 83.2%, and LC values were 25.1% vs 6.7% and 22.4%, respectively. The high drug loading capability of DLMP3 was further confirmed by comparing with other CD-based nanocarrier systems reported [13]. In general, the EE and LC values of micelles are dependent on the composition of the copolymers [26], the length of the hydrophobic blocks [27], the feed ratio of the drug to copolymer, and the preparation methods, as well as the process conditions of micelles [28]. For DLMP3, the higher EE and LC values may be mainly attributed to the introduction of the HBPCSi segments and β-CD moieties, which possess a topological structure and a molecular cavitiy, respectively. Therefore, the core–shell, multiregion encapsulation function of the nanocarrier was also obtained for DLMP3 (Scheme 1E). Zhuo et al. [29] also found that the EE and LC values of the amphiphilic, linear-hyperbranched, block copolymers were higher than those of the amphiphilic, linear block copolymers with similar copolymer composition.

**Table 2:** Loading capacity comparisons for different nanocarriers.

Sample index	EE^a^, %	LC^b^, %

DLMP1	20.5	6.7
DLMP2	83.2	22.4
DLMP3	88.9	25.1

^a^Encapsulation efficiency, determined by UV–vis. ^b^Loading content, determined by UV–vis.

**Figure 3:**
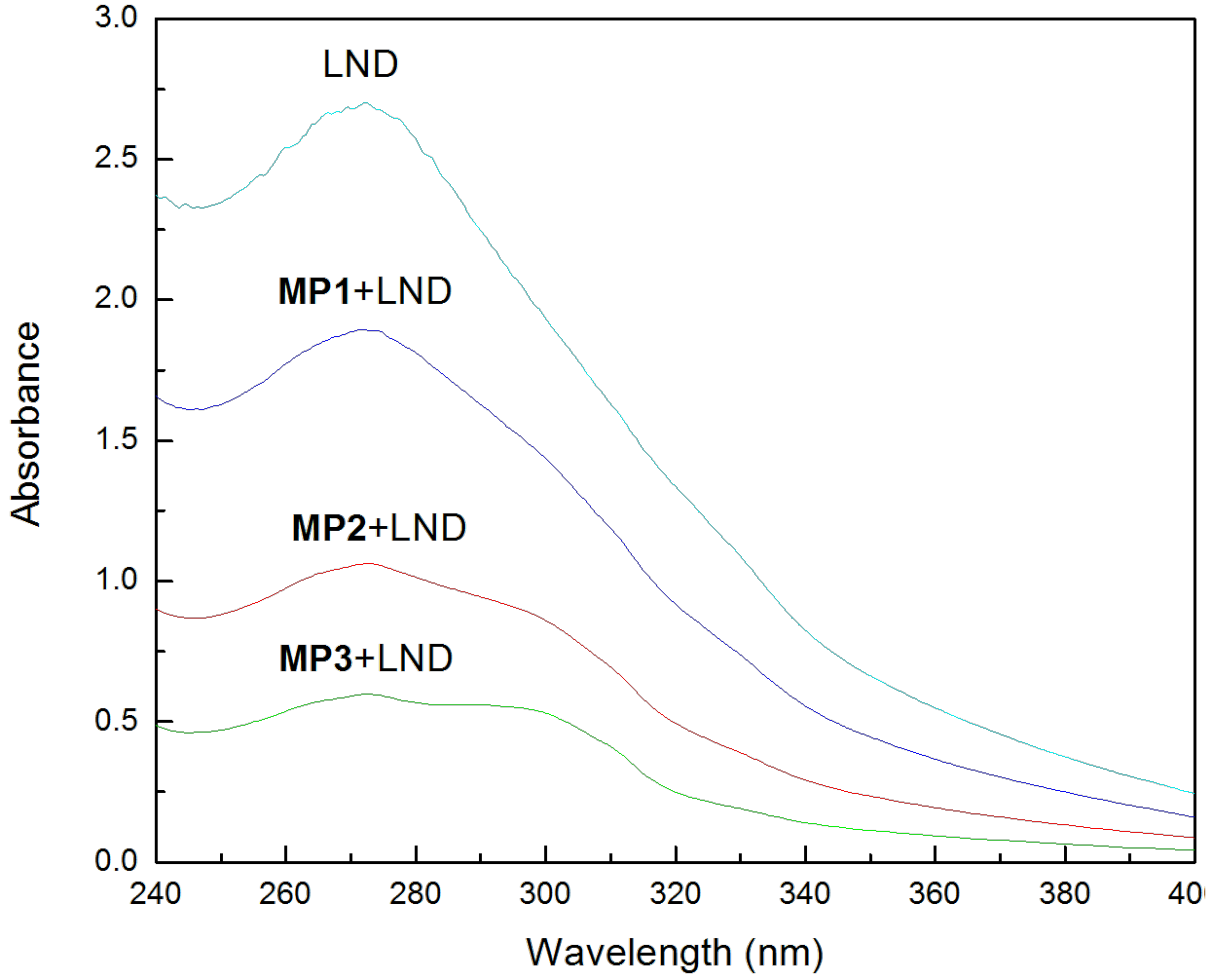
UV–vis spectra of LND solutions in the presence of MP1, MP2 and MP3 in buffer solutions.

UV–vis spectrophotometry was employed to confirm the multiregion encapsulation ability of theamphiphilic polymers [30]. Figure 3 presents the UV–vis spectra of the LND solution in the presence of MP1, MP2 and MP3 in buffer solution. Obviously, the peak intensity of the LND solutions gradually decreased at 272 nm when the samples changed from MP1 to MP2, and finally to MP3 at the same concentration. In general, the encapsulation between the host and guest molecule will occur when the peak intensity of the UV–vis spectrum decrease regularly after the addition of the host; meanwhile, the decreased peak intensity can also reflect the increased encapsulation ability and different encapsulation structures of the host. Therefore, the encapsulation abilities of these micelles are as follows: MP1 < MP2 < MP3. The encapsulation space of MP1 originates only from the core layer of the micelles, whereas for MP2, this arises from the topological structure of the HBPCSi cavities and the core layer of the micelles. For MP3, the synergetic effects of the HBPCSi topology, the core layer of the micelles, and the β-CD cavities can contribute to the encapsulation process.

**Figure 4:**
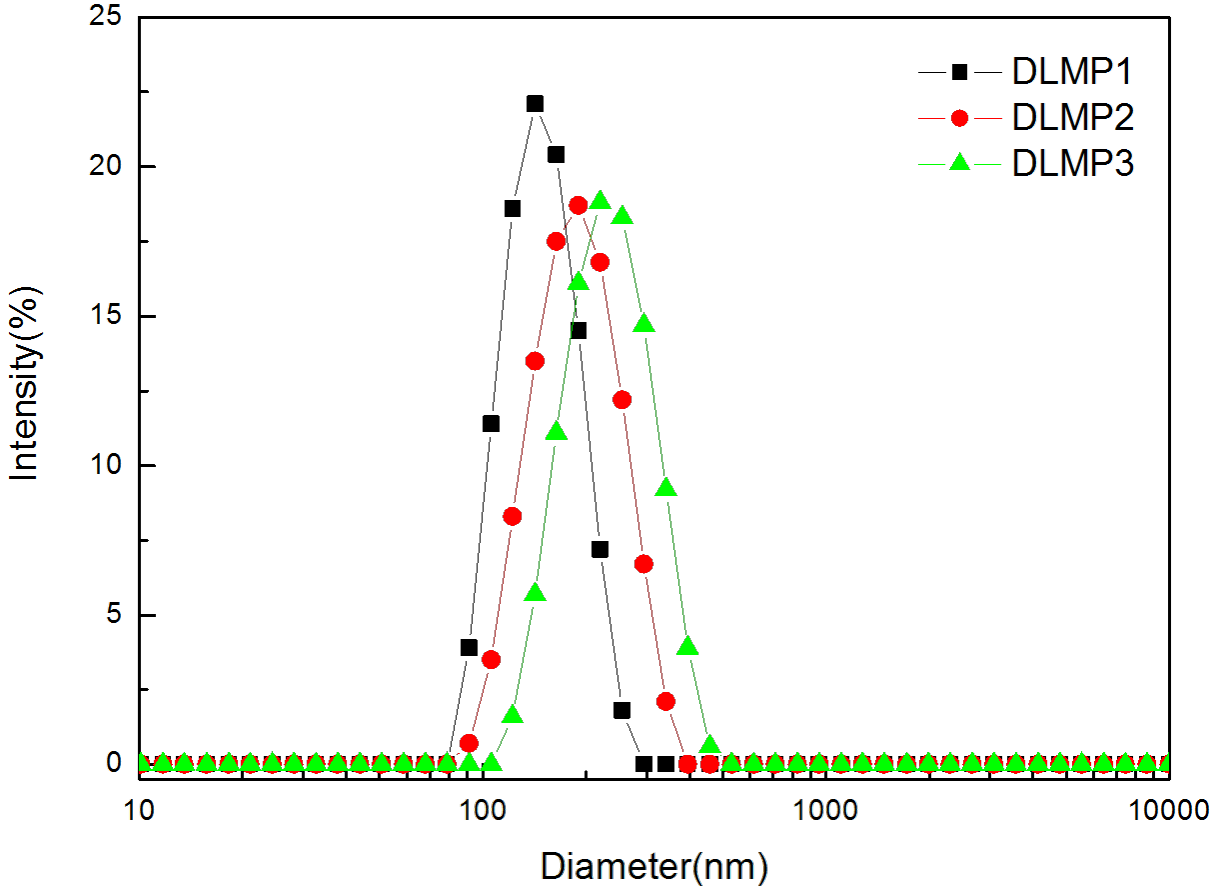
Particle size distributions of DLMP1, DLMP2, and DLMP3 at 25 °C.

**Figure 5:**
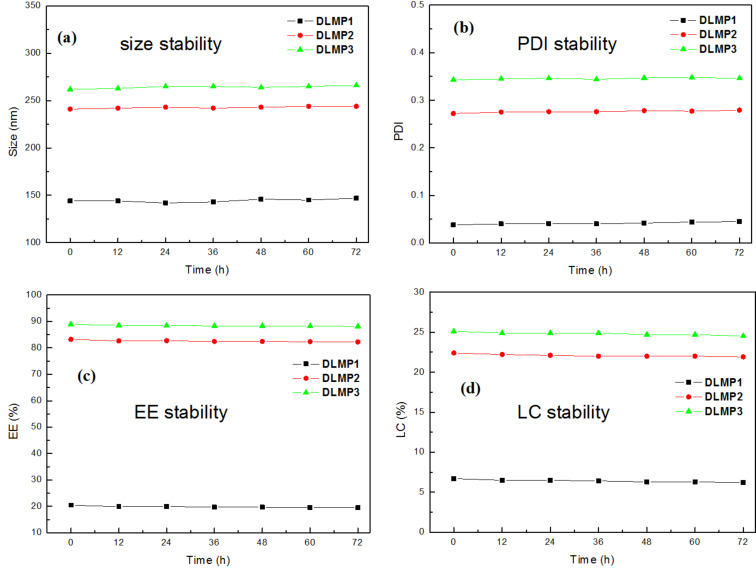
Stability investigation of DLMP1, DLMP2, and DLMP3.

Interestingly, the dimensions of the LND-loaded micelles became slightly smaller and more uniform with a decrease in the PDI, as compared to that of the unloaded micelles (Figure 4 and Table 1). Shen et al. [31] reported a similar phenomenon concerning the change in uniformity of particles in DOX·HCl-loaded nanocapsules. However, the smaller size of LND-loaded micelles was observed for the first time. This may be attributed to the hydrophobic interaction and the electrostatic attraction between LND molecules and PHEMA or HBPCSi segments, resulting in the volume compression and the decrease in size of the LND-loaded micelles. Regardless, the smaller size and the more uniform distribution are beneficial for the application of MP1–MP3 as multifunctional nanocarriers. In addition, the stability of DLMP1–DLMP3 still must be tested [32]. As shown in Figure 5, no significant change was found in the diameter, PDI, EE and LC within 72 h. These results indicated that they are stable in buffer solution for an extended period.

**Figure 6:**
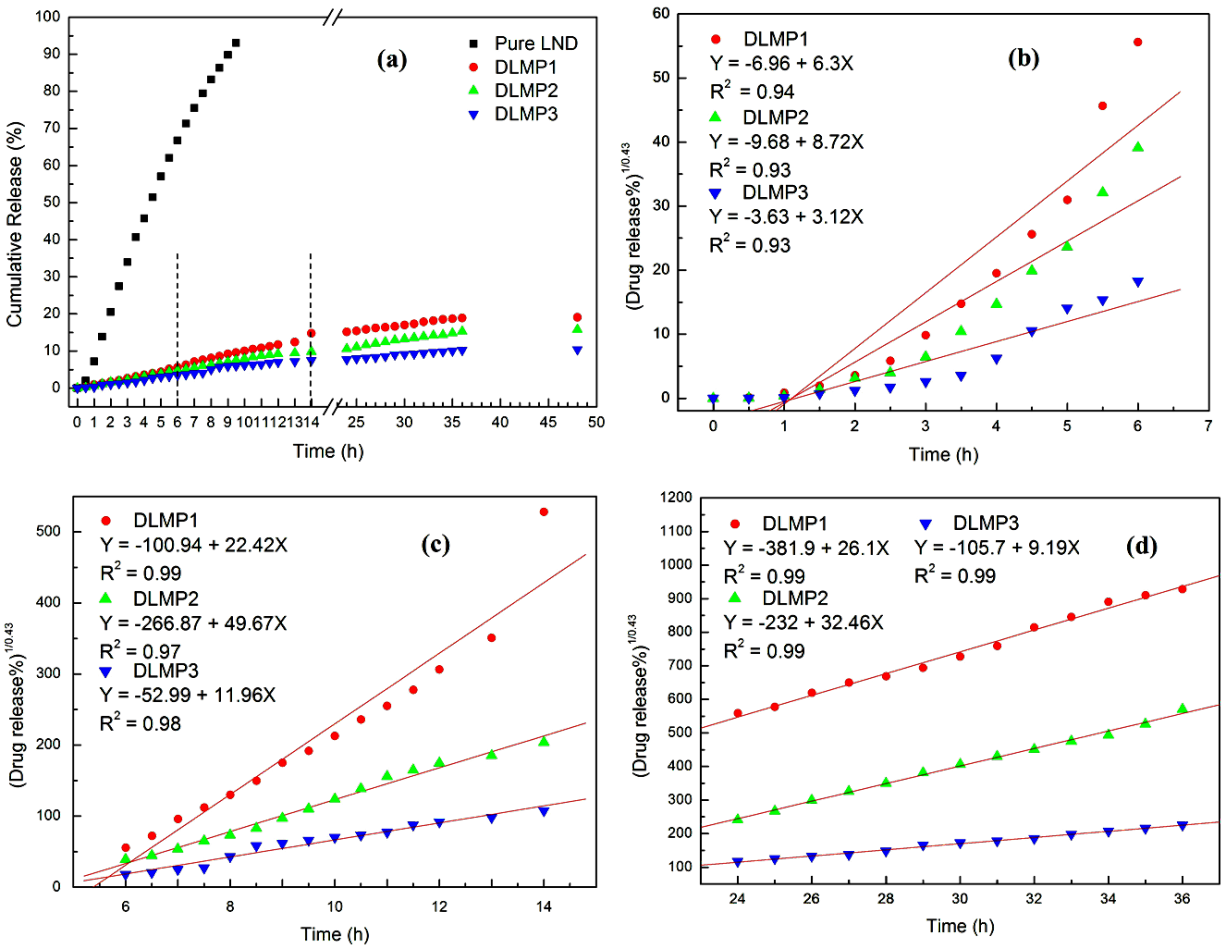
Release profiles (a) and release kinetics (b–d) of LND from DLMP1, DLMP2 and DLMP3 in buffer solution at 37 °C.

To test the controlled release behavior of LND-loaded micelles, the release experiments were carried out by dialysis. Figure 6a shows the release profiles of LND from LND-loaded micelles at 37 ºC. Compared with pure LND (without micelles), no significant burst release was observed from LND-loaded micelles. This result indicates that the function of controlled release was obtained by taking advantage of the stimuli-responsive property of the PDMAEMA segments. Furthermore, the cumulative amounts of LND released from DLMP1, DLMP2 and DLMP3 were only 18.4, 15.5, and 9.2%, respectively, within the release time of 48 h, indicating the clear sustained release behaviour. A similar sustained release phenomenon was also observed in other high drug loading, multifunctional nanocarriers [31]. More importantly, it can be seen from Figure 6a that the sustained release ability of DLMP3 was enhanced as compared with DLMP1 and DLMP2 after the release of 6 h. For example, their cumulative release amounts were 11.6, 9.5, and 6.8%, respectively, within 14 h. This may be attributed to the introduction of the HBPCSi segments and β-CD moieties. Generally, the hydrophobic–hydrophobic interactions between drug molecules and hydrophobic core layer of micelles make an important contribution to the sustained release of drugs from micelles [32,34]. In our micelle systems, the hydrophobicity of the core layer of MP2 was enhanced with the introduction of the HBPCSi segments, followed by the enhancement of hydrophobic–hydrophobic interactions between LND molecules and the core layer. As a result, DLMP2 had a stronger sustained release behaviour than DLMP1. Other researchers also proved that the amphiphilic, linear-hyperbranched, block copolymers had more sustained drug release behavior than those of amphiphilic, linear block copolymers [21]. Furthermore, the β-CD moieties of the shell layer of MP3 may form inclusion complexes with LND and further retard its release [33]. Therefore, DLMP3 possesses the strongest sustained release capability among these LND-loaded micelles.

For further comprehension of the sustained release behaviours, the corresponding release mechanism of LND from DLMP1, DLMP2 and DLMP3 was determined by investigating their release kinetics. As can be seen from Figure 6a, there are three main stages in the complete release process of DLMP1, DLMP2 and DLMP3, ranging from 0 to 6 h, 6 to 14 h, and 24 to 36 h, respectively. Therefore, kinetic equations were first derived according to the method reported (see Experimental Section) [34–36]. Figure 6b–d shows the kinetic release profiles. A good linear correlation was found between the cumulative release amount and release time within the given time range, as evidenced by the R^2^ values which were close or equal to 0.99. Therefore, it could be concluded that the release mechanism of LND-loaded micelles agrees with the “Fickian diffusion” behavior, which is mainly described by a diffusion-controlled mechanism [34]. The study of the release mechanism will be useful to determine the pharmacokinetics of nanocarriers in the future.

## Conclusion

In conclusion, we demonstrated a novel ABC employed as a multifunctional nanocarrier with high loading, multiregion encapsulation, and controlled release capability. These obtained micelles possess improved drug loading capability as compared to their counterparts due to the introduction of HBPCSi segments. They can simultaneously encapsulate drug molecules not only in the hydrophobic core layer, but also in the hydrophilic shell layer by taking advantage of the β-CD cavities. They also exhibit a controlled drug release ability based on the diffusion release mechanism.

## Experimental

Experimental details can be found in the Supporting Information.

## Supporting Information

File 1 Experimental details, synthetic routes, and characterization results of P1–P3.
