# Supplementary material for: Linear-g-hyperbranched and cyclodextrin-based amphiphilic block copolymer as a multifunctional nanocarrier
Source: Beilstein J Org Chem. 2014 Nov 18;10:2696–703. doi: 10.3762/bjoc.10.284 (PMC4273233; doi:10.3762/bjoc.10.284)
Supplement: File 1 — Experimental details, synthetic routes, and characterization results of P1–P3. [file Beilstein_J_Org_Chem-10-2696-s001.pdf]

Supporting Information

for

Linear-*g*-hyperbranched and cyclodextrin-based amphiphilic block copolymer as a multifunctional nanocarrier

Yamei Zhao[1], Wei Tian*[2], Guang Yang[2] and Xiaodong Fan[2]

Address:[1]College of Environmental and Chemical Engineering, Xi'an Polytechnic University, Xi'an, 710048, P. R. China, and [2]The Key Laboratory of Space Applied Physics and Chemistry, Ministry of Education and Shanxi Key Laboratory of Macromolecular Science and Technology, School of Science, Northwestern Polytechnical University, Xi'an, 710072, P. R. China

Email: Wei Tian - happytw_3000@nwpu.edu.cn
*Corresponding author

Experimental details, synthesis routes, and characterization results of P1–P3

Table of Contents

1. **Experimental details**

2. **Synthesis and characterization of amphiphilic triblock polymer (P1)**

3. **Synthesis and characterization of amphiphilic triblock polymer with hyperbranched polycarbonsilane (P2)**

4. **Synthesis and characterization of amphiphilic triblock polymer with hyperbranched polycarbonsilane and β-cyclodextrin moieties (P3)**

5. **References**

1. Experimental details

Materials

S,S'-bis(α α,'-dimethylacetic acid) trithiocarbonate (BDATTC), methyldiallylsilane (AB_2 monomer), and monoiodide-substituted *β*-CD (mono-6-I-*β*-CD) were synthesized according to the methods reported in the literature, respectively [S1-S3]. 2-hydroxyethyl methacrylate (HEMA) and *N,N*-dimethylaminoethylm ethacrylate (DMAEMA) were purchased from ACROS Chemical Industries (USA), and purified by distillation under reduced pressure. Acryloyl chloride (AC) was purchased from Haimen Best Fine Chemical Ltd (Haimen, China). (Dimethylamino)-pyridine (DMAP) was purchased from Zhejiang Jintan Chemical Plant (Jintan City, Zhejiang Province, China). Chloroplatinic acid (H_2PtCl_6) (39%, platinum) was provided by Shaanxi Kaida Chemical Ltd (Xi'an City, Shaanxi Province, China). 2,2'-azobisisobutyronitrile (AIBN) was purchased from Tianjiin Kermel Chemical Reagents Development Center (Tianjin City, China), and was recrystallized twice from ethanol before use. Lonidamine (LND) was purchased from Changzhou Ruiming Pharmaceutical Ltd. (>98% purity) (Changzhou City, China). Others reagents were all purchased from Tianjiin Kermel Chemical Reagents Development Center (Tianjin City, China). All reagents were dried with 4 Å grade molecular sieves before use without further purification.

Measurements

The Fourier transform infrared spectroscopy **(**FTIR) spectra were obtained on a WQF-31 spectrometer (Rui Li, Beijing, China) by forming samples into thin films on KBr. Spectra were collected in transmission mode with the wavenumber range set from 4000 to 400 cm^{-1} (resolution 1 cm^{-1}, measuring time 300 s).

The ^{1}H nuclear magnetic resonance (NMR) and ^{13}C NMR spectra were conducted on a Bruker Avance 300 spectrometer (Bruker BioSpin, Switzerland) operating at 300 MHz (^{1}H) in d_6-DMF.

The molecular structure parameters of the resulting polymers were determined on a DAWN EOS size exclusion chromatography/multiangle laser light scattering (SEC/MALLS) instrument equipped with a viscometer (Wyatt Technology, USA), and HPLC grade DMF containing LiCl (0.01 mol/L) (at 40 ºC) was used as the eluent at a flow rate of 0.5 mL/min. The chromatographic system consisted of a Waters 515 pump, differential refractometer (Optilab rEX), and one column, MZ 10^3 Å 300 × 8.0 mm. A MALLS detector (DAWN EOS), a quasielastic light scattering (QELS), and a differential viscosity meter (ViscoStar) were placed between the SEC and the refractive index detector. The molecular weight (M_w) and molecular weight distribution (MWD) were determined by a SEC/DAWN EOS/OptilabrEX/QELS model. ASTRA software (Version 5.1.3.0) was utilized for acquisition and analysis of data.

The particle size and particle size distribution index (PDI) were measured by a Zetasizer Nano-ZS dynamic light scattering (DLS) (Malvern Instruments, UK) device. Samples were kept at equilibrium at a predetermined temperature for 5 min before data collection and were measured at 25 ºC.

Transmission electron microscopy (TEM) experiments were carried out on a JEOL JEM-3010 instrument (JEOL, Japan). The mMicelle solutions were dropped onto copper grids and dried at room temperature before measurement.

UV–vis spectrophotometry measurements were preformed on a Shimadzu UV-2550 model spectrophotometer (Shimadzu, Japan). All solutions were kept at 37 ^{o}C for 48 h prior to measurements.

Synthesis of macro chain transfer agent (PHEMA-macroCTA) (Similar as described in Ref. S4)

In a flask equipped with magnetic stirring, BDATTC (141 mg, 0.5 mmol) and AIBN (2.46 mg, 0.015 mmol) were first dissolved in tertbutyl alcohol (5 mL), and then HEMA (5.8 g, 0.05 mol) was added under vigorous stirring. The mixture was degassed under vacuum for 15 min, and then purged under dry nitrogen for 30 min. The procedure described above was repeated three

times. The flask was then sealed under vacuum. Polymerization was conducted at 80 °C for 10 h, and then the system was cooled down to ambient temperature. The final product was precipitated in cold methanol and dried under vacuum at 40 °C for 5 d.

Synthesis of triblock copolymer (PHEMA-*b*-PDMAEMA-*b*-PHEMA) (Similar as described in Ref. S4)

PHEMA-macroCTA (0.1 g) and AIBN (2.46 mg, 0.015 mmol) were first dissolved in DMF (4 mL), and then DMAEMA (7.9 g, 0.05 mol) was added under vigorous stirring. The mixture was degassed under vacuum for 15 min, and then purged under dry nitrogen for 30 min. The procedure described above was repeated three times. The flask was then sealed under vacuum. Polymerization was conducted at 80 °C for 2 h, and then the system was cooled down to ambient temperature. The solvent was evaporated under reduced pressure at 80 °C/10^{-4} bar, and the raw product obtained was again dissolved in 1,4-dioxane. The final product was precipitated in cold hexane and dried under vacuum at 40 °C for 5 d.

Synthesis of P1 (Similar as described in Ref. S3)

PHEMA-*b*-PDMAEMA-*b*-PHEMA (1.23 g), THF (50 mL), pyridine (10.81 g, 0.14 mol) and DMAP (10 mg) were mixed in a 500 mL three-neck round-bottomed flask with a thermometer in an ice water bath. Under vigorous stirring, AC (12.38 g, 0.14 mol) dissolved in THF (20 mL) was added dropwise to the mixture. The pyridinium hydrochloride could be observed as a white precipitate at an early stage, and then later changed to a yellowish solid. The reaction was then kept at ambient temperature for 24 h, and the precipitate was filtered out and the raw product was left in the filter. The solvent was evaporated under reduced pressure at 50 °C/10^{-4} bar. The final product was precipitated in cold diethylether and dried under vacuum at ambient temperature for 5 d.

Synthesis of P2

P1 (0.97 g), and DMF (2 mL) solvents were first mixed in a 100 mL round-bottomed flask equipped with a funnel under nitrogen atmosphere. Then, the first generation AB_2 monmer (0.13 g) dissolved in DMF (1 mL) was added dropwise into the solution under vigorous

stirring. A H_2PtCl_6 divinyltetramethyldisiloxane solution was used as the catalyst for the hydrosilylation addition reaction. The system was then moved to an oil bath at 50 ^oC under vigorous stirring until no Si-H groups were detected by FTIR (2136 cm^{-1}). The second generation AB_2 monmer (0.26 g) (which was dissolved in DMF (1 mL)) was added again, and the same method was used to detect whether the reaction was finished. After the system was cooled down to ambient temperature, the mixture was precipitated in cold methanol, and then the precipitate as a cyclic product was filtered out, while the raw product was left in the filter. The solvent was evaporated under reduced pressure at 40 $^oC/10^{-4}$ bar. The final product was precipitated in cold diethylether and dried under vacuum at ambient temperature for 5 d.

Synthesis of P3 (Similar as described in Ref. S3)

P2 (0.3 g) and mono-6-I-*β*-CD (0.6 g) were first dissolved in dry DMF (3 mL), and then the system was kept under vigorous stirring at 90 ^oC in an oil bath for 72 h. After cooling down to ambient temperature, the mixture was dialyzed in a dialysis bag (molecular weight cut off: 3500) against distilled water for 7 d. It was refreshed at an interval of 5 h. The final product was lyophilized and kept in glassware under vacuum for further use.

Preparations of P1, P2 and P3 micelles (Similar as described in Ref. S5)

Preparations of **P1, P2** and **P3** micelles were carried out at room temperature using DMF and water as solvents. **P1** (or **P2**, **P3**) (20 mg) was first dissolved in DMF (1mL), and then distilled water (20 mL) was added dropwise to the solution. The system was continuously stirred for 16 h. Later, the mixture was subjected to dialysis (molecular weight cut off: 3500) against water for 2 d. When the dialysis was complete, the micelle solution was subjected to centrifugation for the removal of larger aggregates. The volume of the obtained micelle solutions was about 23–25 mL, and they were designated as **P1, P2** and **P3** micelles, respectively.

Encapsulation behavior of P1, P2 and P3 micelles

The encapsulation behavior of **P1, P2** and **P3** micelles was measured by UV-vis spectrophotometry, using LND (5×10^{-5} mol/L) as a guest molecule in a buffer solution with

ionic strength equal to 0.1 mol/L and pH = 7.4. Typically, the encapsulation procedures were similar to the preparation of micelles, and no further change were made except for using LND as a buffer solution instead of the distilled water. All solutions were maintained for more than 12 h to ensure binding equilibrium and then stirred prior to measurement.

Drug loading

P1 (or **P2**, **P3**) (20 mg) and LND (2 mg) were dissolved in DMF (1 mL) under vigorous stirring, and the subsequent procedures of preparing LND-loaded micelle solutions denoted as **DLMP1** (or **DLMP2**, **DLMP3**) were the same as those for preparing polymer micelles. The encapsulation efficiency (EE) and loading content (LC) of LND were calculated by using Equations 1 and 2 as follows:

$$EE = W_{\text{drug in miclles}}/W_{\text{feeding drug}} \times 100\% \quad (1)$$

$$LC = W_{\text{drug in miclles}}/W_{\text{freeze-dried micelles}} \times 100\% \quad (2)$$

where $W_{\text{drug in miclles}}$ was determined by UV–vis spectroscopy. Here, 1 mL of **DLMP1** (or **DLMP2**, **DLMP3**) was diluted to 10 mL with DMF, and the LND concentration was determined by measuring the absorbance at 298.5 nm.

Controlled drug release in vitro (Similar as described in Ref. S4)

DL-P1 (or **DL-P2**, **DL-P3**) (10 mL) was sealed using a dialysis bag of 4 cm in length (molecular weight cut off: 3500). The bags were immersed into 40 mL of a buffer solution with ionic strength equal to 0.1 mol/L and pH = 10 at 37 ^{o}C. At given time intervals, 5.0 mL of buffer solution was withdrawn and replaced with 5.0 mL of fresh. The concentration of the released LND was analyzed by measuring the absorption at 298.5 nm. All release measurements were carried out in triplicate for each sample, and an average value was adopted. The cumulative release was calculated by using Equation 3 as follows:

$$\text{Cumulative release (\%)} = \frac{100\times(40.0C_n + 5.0\sum C_{n-1})}{W_0} \quad (3)$$

where W_0 (mg) is the weight of the LND in **DLMP1** (or **DLMP2**, **DLMP3**), C_n (mg/ml) is the concentration of the LND in buffer solution (which was withdrawn n times), and C_{n-1} (mg/ml) is the concentration of the LND in buffer solution (which was withdrawn n-1 times).

Release kinetics (Similar as described in Ref. S5)

The release kinetics of **DLMP1**, **DLMP2**, and **DLMP3** were determined by a simple semi-empirical equation (Equation 4) and a modified Equation 5 used to describe the release behavior of polymeric micelles [S6-S8].

$$\frac{M_t}{M_\infty} = \mathrm{kt^n} \qquad (4)$$

$$(\mathrm{release\%})^{1/n} = (100 \times (\frac{M_t}{M_\infty}))^{1/n} = k'\,\mathrm{t} \qquad (5)$$

where M_t and M_∞ are the cumulative amount of released LND at time t and infinite time, respectively; k is the release constant while k' is the constant proportional to k; the exponent n describes the kinetic and the release mechanism. For a diffusion–degradation controlled release system, n for spherical particles is usually between 0.43 and 0.85. When n is close to 0.43, diffusion is the major driving force, which is called the "Fickian diffusion." When n is close to 0.85, the release is mainly controlled by degradation [S7,S8]. In order to obtain a linear fit for the drug release data, Equation 5 rearranged from Equation 1 is more suitable for this release system, and 0.43 should be used as the n value in our micelle systems [S6,S8].

2. Synthesis and characterization of amphiphilic triblock polymer (P1)

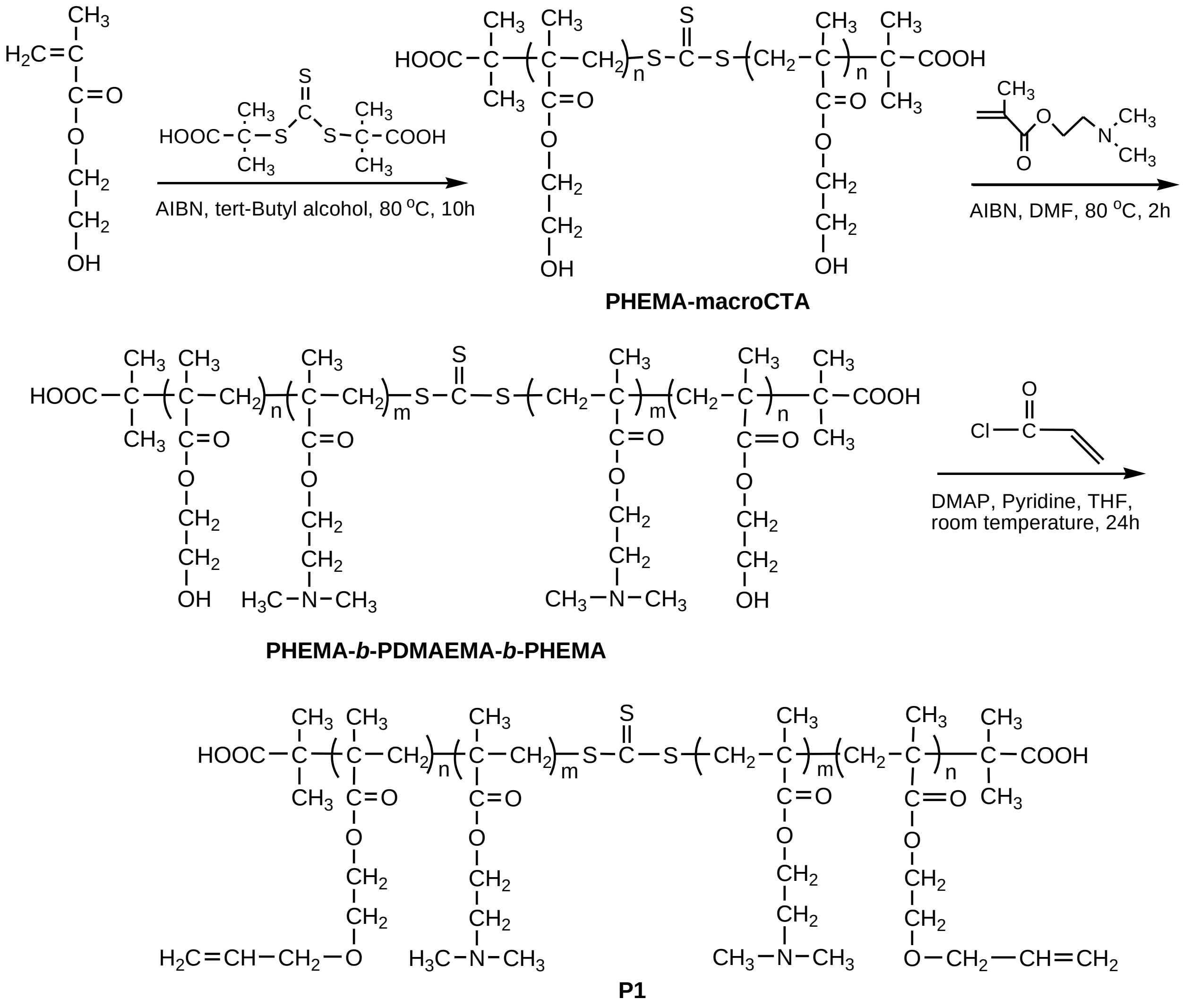

Scheme S1. Synthetic routes for **P1**

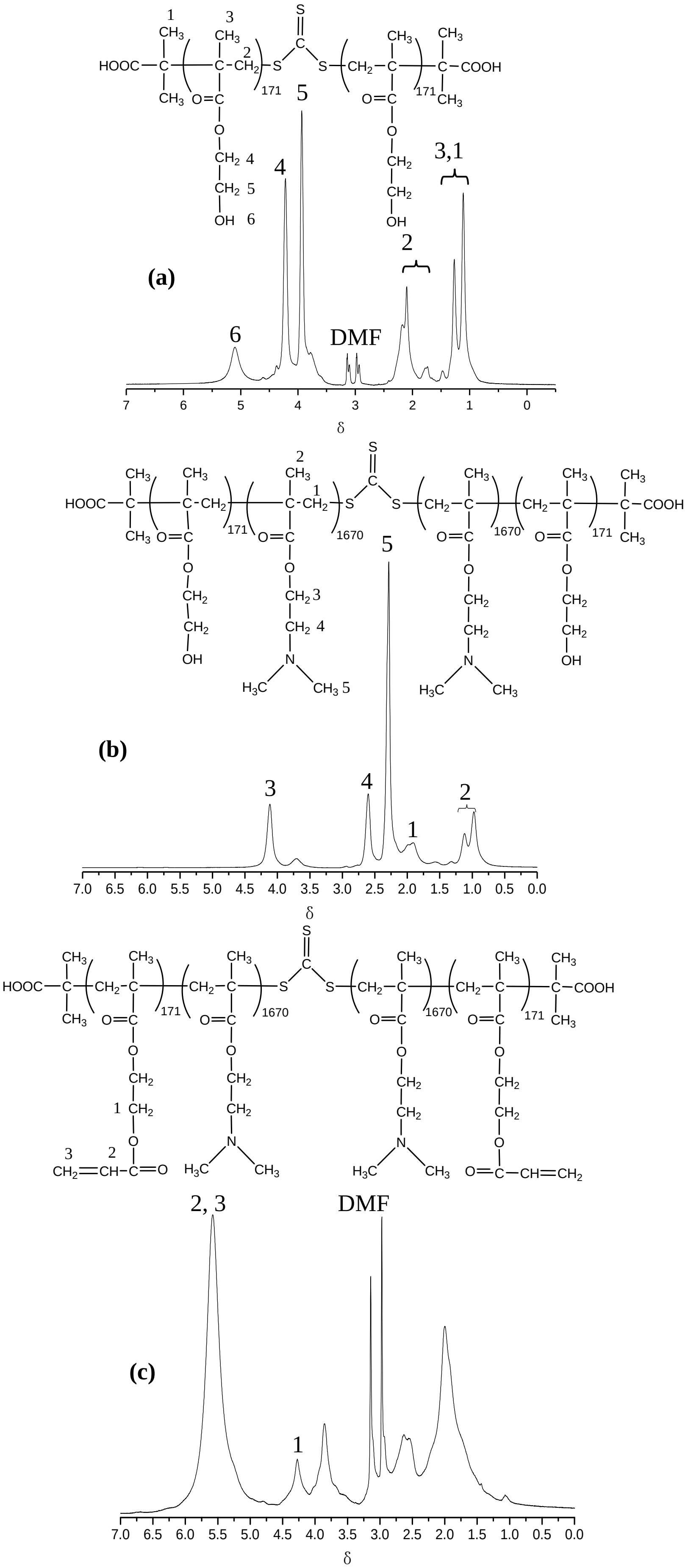

Figure S1. ^{1}H NMR spectra of PHEMA-macroCTA (a), PHEMA-*b*-PDMAEMA-*b*-PHEMA (b) and **P1** (c)

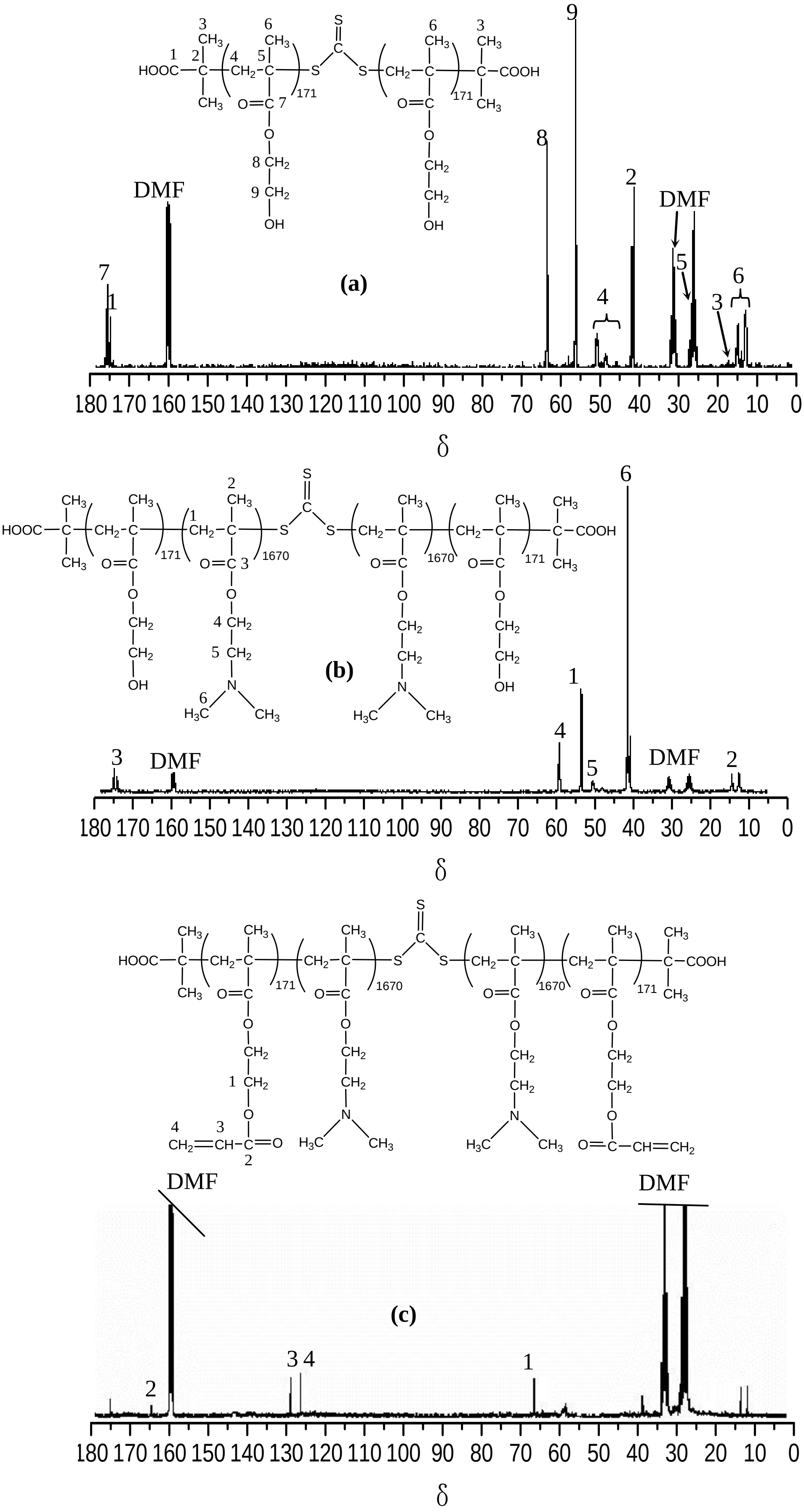

Figure S2. ^{13}C NMR spectra of PHEMA-macroCTA (a), PHEMA-*b*-PDMAEMA-*b*-PHEMA (b) and **P1** (c)

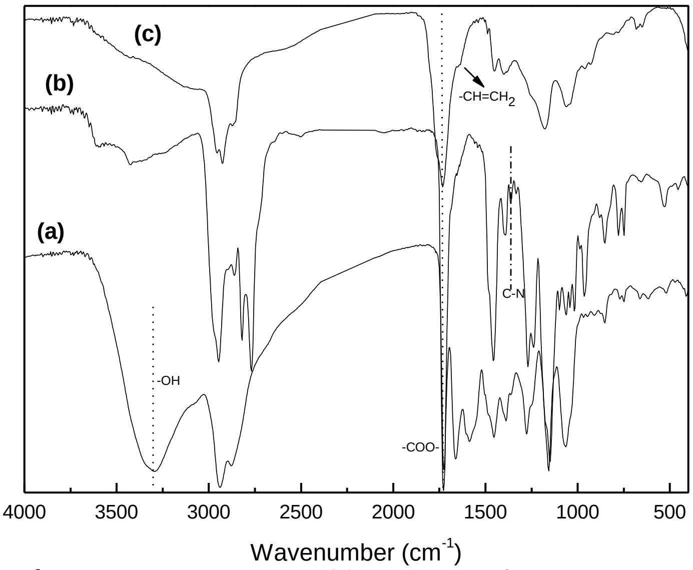

Figure S3. FTIR spectra of PHEMA-macroCTA (a), PHEMA-*b*-PDMAEMA-*b*-PHEMA (b) and **P1** (c)

As can be seen from Figures S1-S3, all the protons and carbon atoms of PHEMA-macroCTA and PHEMA-*b*-PDMAEMA-*b*-PHEMA can be clearly assigned. It should be pointed out that most proton and carbons signals of PHEMA-macroCTA can no longer be detected in Figure S1b and Figure S2b, indicating the high polymerization efficiency and high molecular weight of PHEMA-*b*-PDMAEMA-*b*-PHEMA. Furthermore, the appearance of new chemical shifts in the ^{1}H NMR spectrum (Figure S1c) at δ 5.56 (—CH=CH_2) and the ^{13}C NMR spectrum (Figure S2c) at δ 126.8 (—CH_2=CH_2), δ 129.5 (—CH_2=CH_2) and δ 164.8 (—COO—) indicate that the acylation reaction was successfully carried out. Additional evidence comes from FTIR spectra (Figure S3c) where the peak found at 1640 cm^{-1}, assigned to double bond absorption, appears after the grafting reaction, further confirming the acryloyl chloride was successfully grafted onto PHEMA-*b*-PDMAEMA-*b*-PHEMA.

Table 1: Molecular structure parameters of resulting polymers.

Sample index	M_n [a]	M_w [a]	MWD [a]	DP [b]
PHEMA-macroCTA	44430	64870	1.46	342 (PHEMA block)
PHEMA-*b*-PDMAEMA-*b*-PHEMA	308200	416000	1.35	1670 (PDMAEMA block)
**P1**	323200	384600	1.19	-
**P2**	377500	554900	1.47	430 (HBPCSi block)
**P3**	422900	896500	2.12	-

[a] Molecular weight and molecular weight distributions, determined by SEC/MALLS.
[b] Average degree of polymerization, determined by SEC/MALLS.

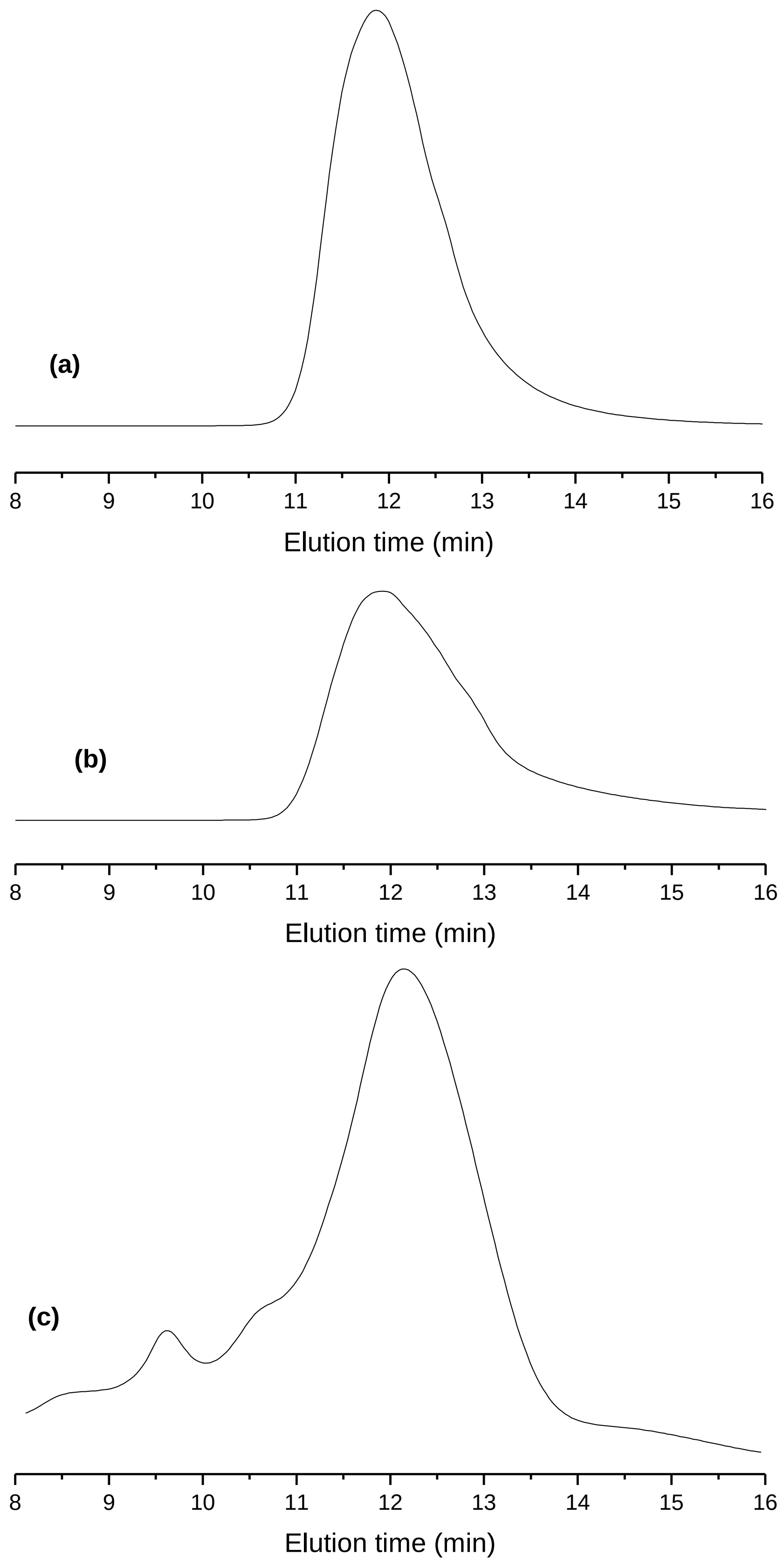

Figure S4. SEC elution cures of PHEMA-macroCTA (a), PHEMA-*b*-PDMAEMA-*b*-PHEMA (b) and **P1** (c)

3. Synthesis and characterization of an amphiphilic triblock polymer with hyperbranched polycarbonsilane (P2)

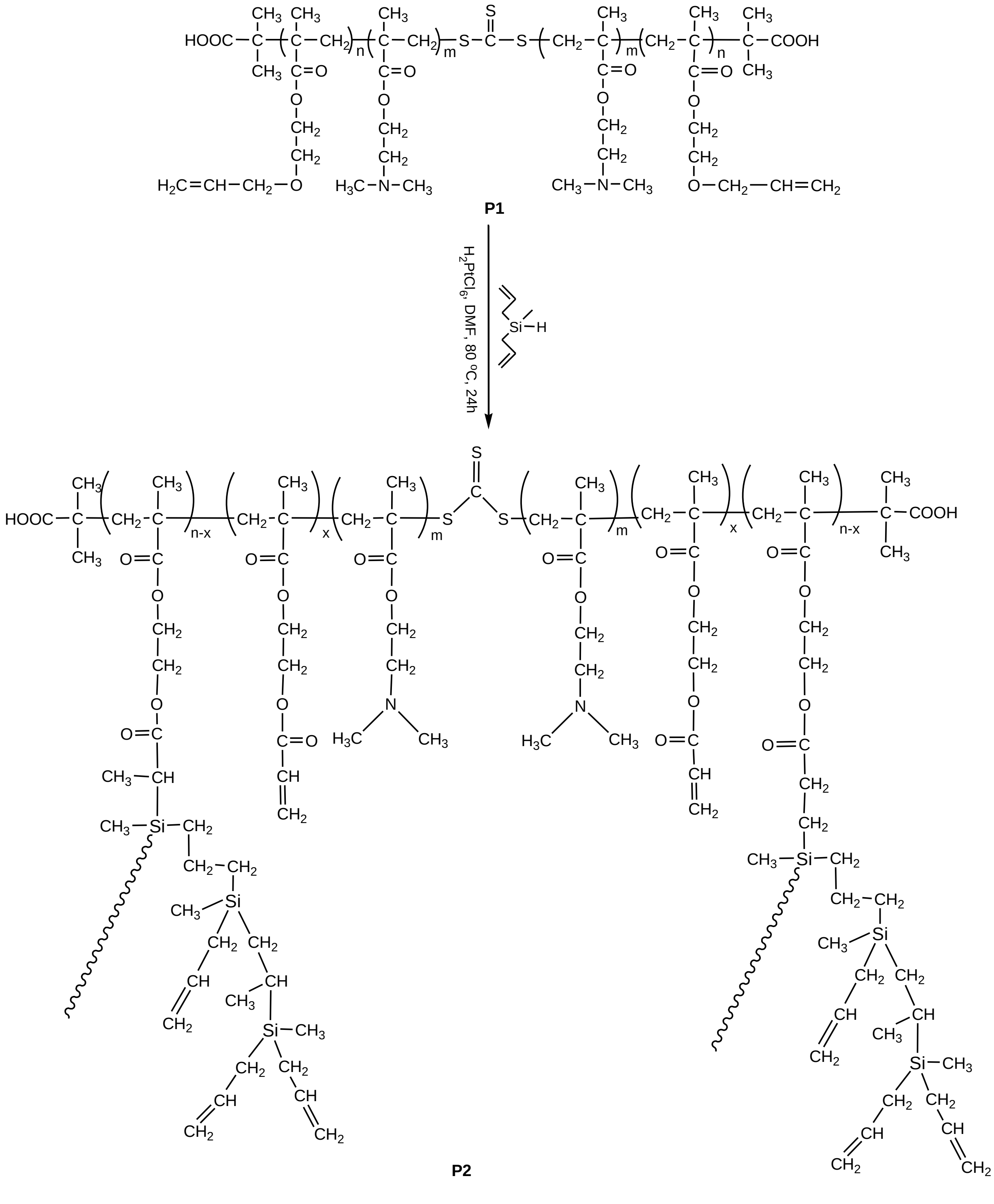

Scheme S2. Synthesis route for **P2.**

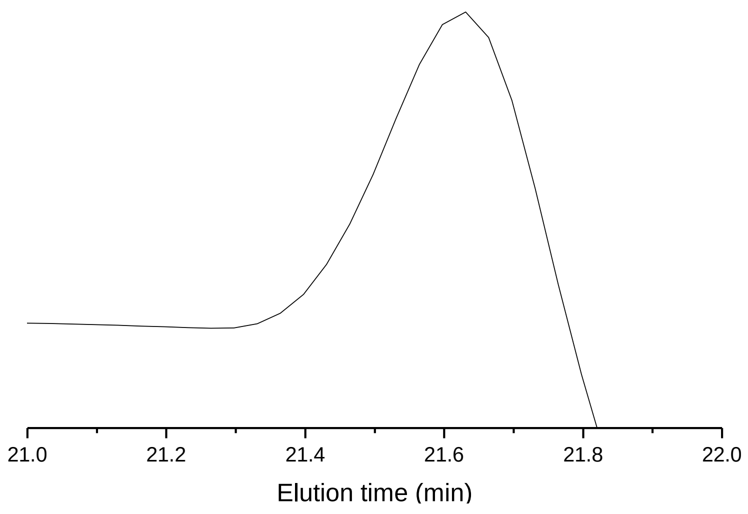

Figure S5. SEC elution cure of **P2**

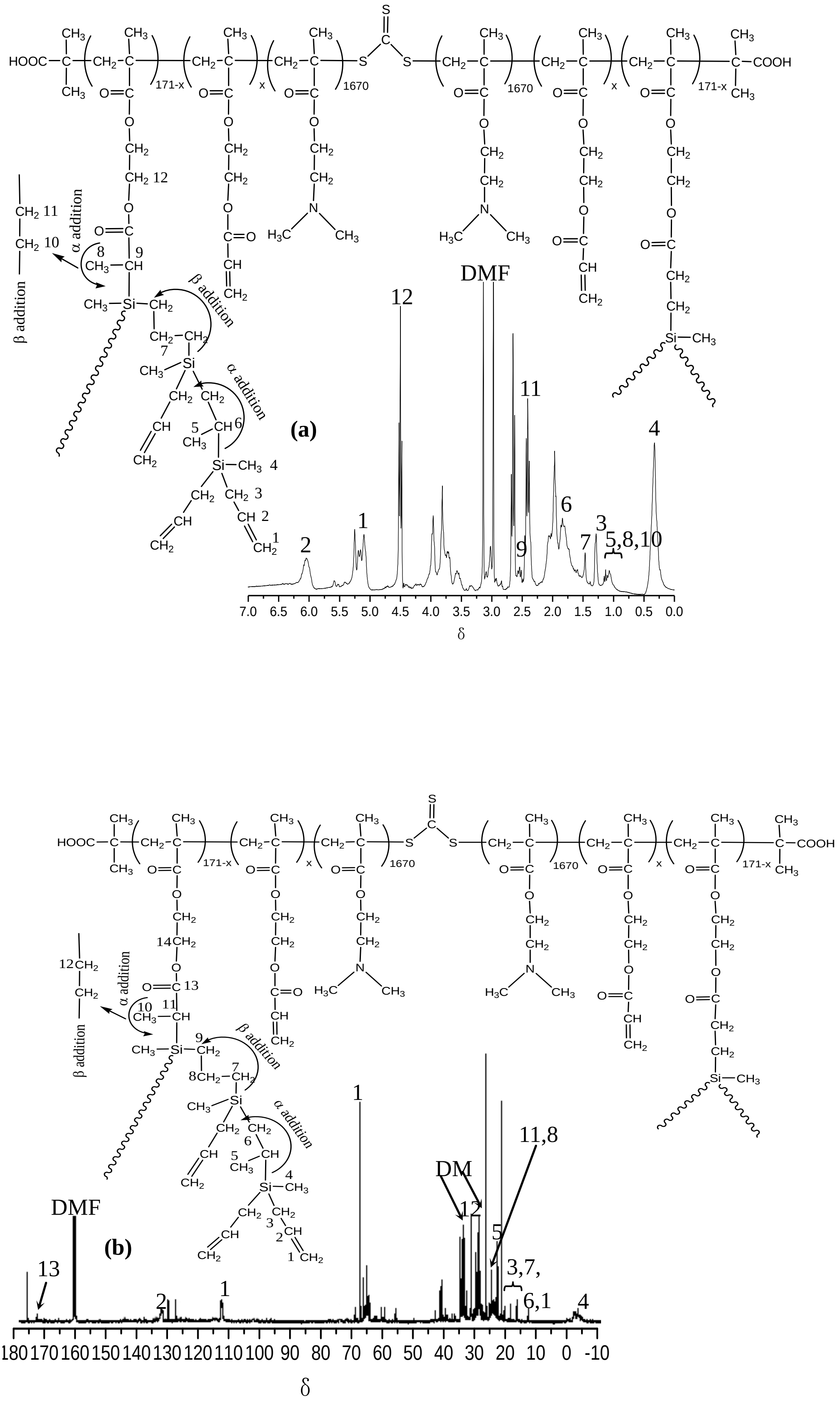

Figure S6. ^{1}H NMR (a) and ^{13}C NMR (b) spectra of **P2**

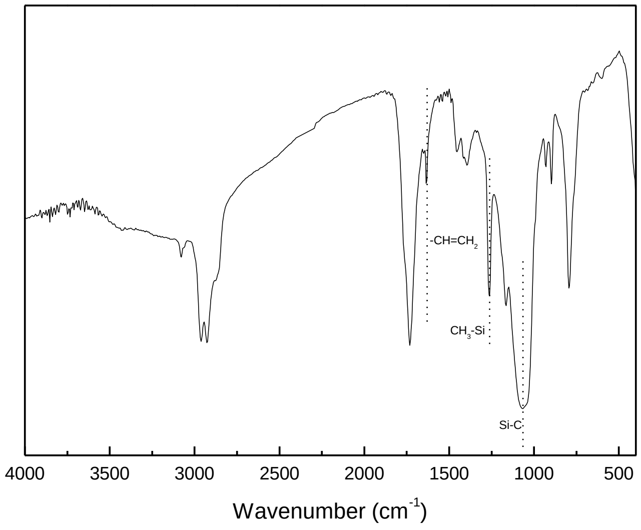

Figure S7. FTIR spectrum of **P2**

From Figure S6a, the appearance of characteristic peaks at δ 0.95–1.22 (—CH_3), 1.51 (—CH_2—) and 1.84 (—CH—) indicates that the α and β additions simultaneously took place during the hydrosilylation reaction according to our knowledge [S10]. The ^{13}C NMR spectrum in Figure S6b further confirmed this point. However, the ^{1}H NMR (δ 5.71) and ^{13}C NMR (δ 127.5, and δ130.2) data proved that the reservation of double bond groups resulting from the incomplete hydrosilylation reaction was probably due to steric hindrance. Frey et al. found the same result during the synthesis of linear-hyperbranched block copolymers consisting of polystyrene and HBPCSi blocks [S11]. Fortunately, the residual double bond groups have little effect on the subsequent micelle formation and drug loading.

4. Synthesis and characterization of an amphiphilic triblock polymer with hyperbranched polycarbonsilane and β-cyclodextrin moieties (P3)

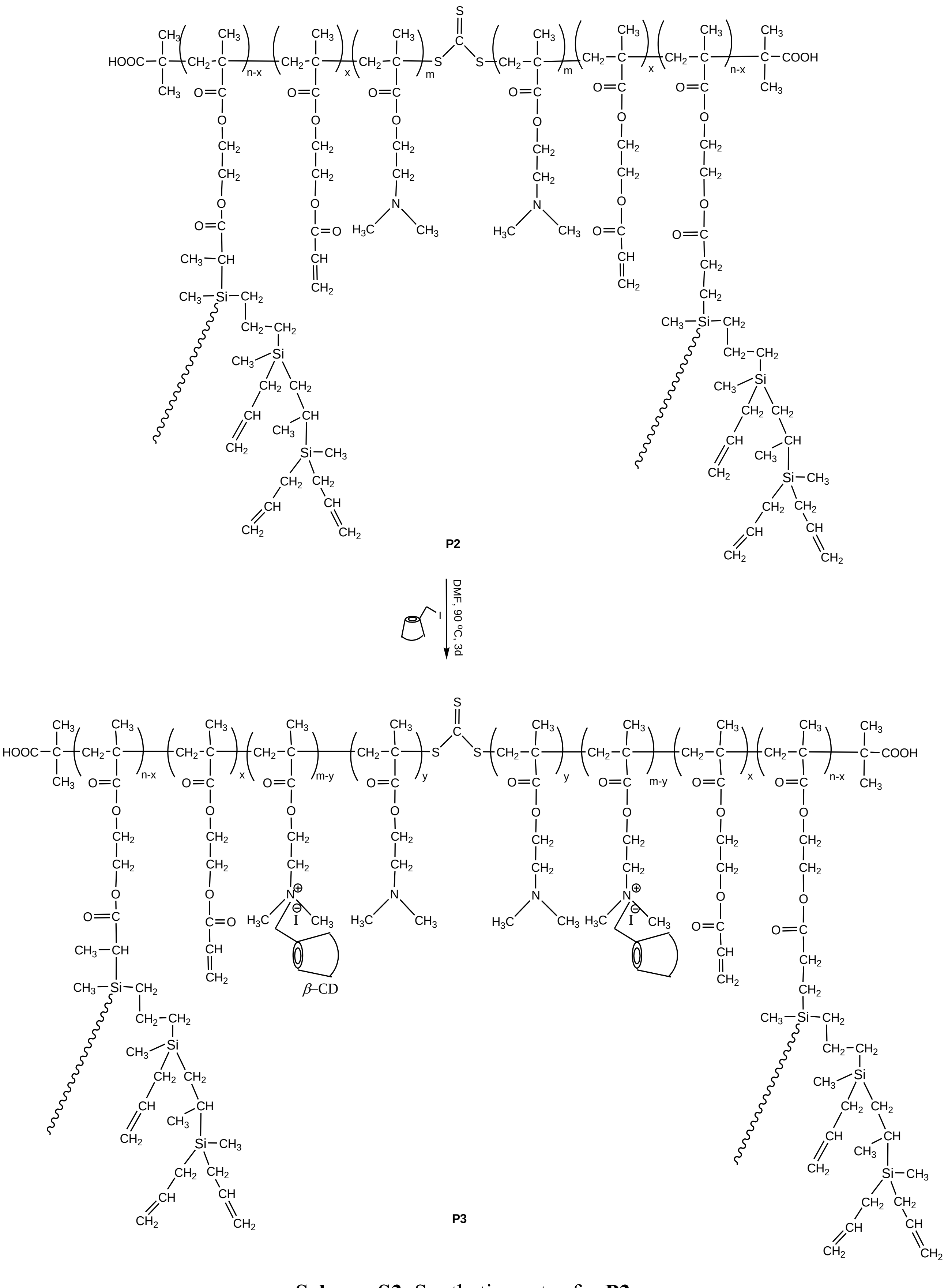

Scheme S3. Synthetic routes for **P3**

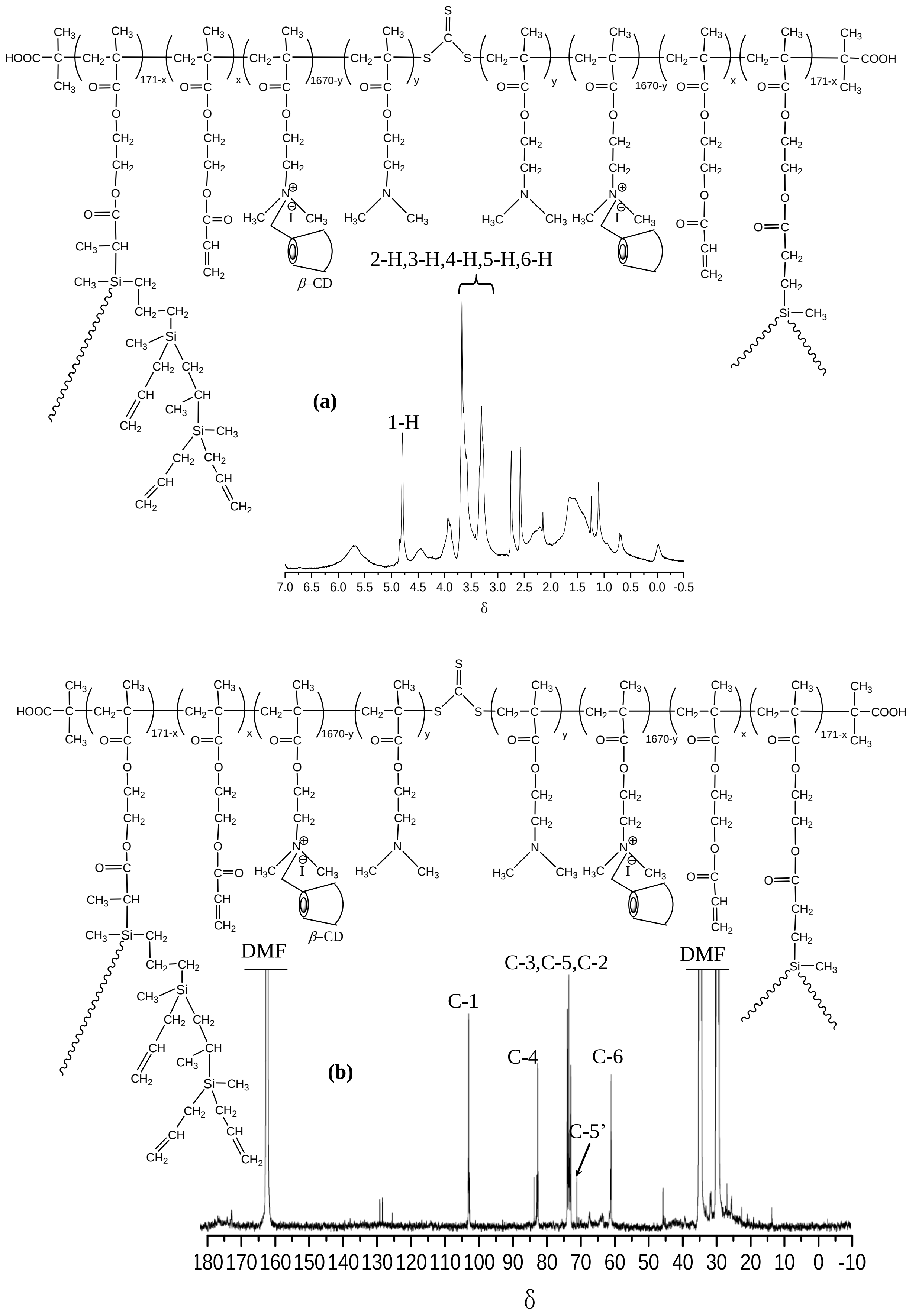

Fgure S8. ^{1}H NMR (a) and ^{13}C NMR (b) spectra of **P3**

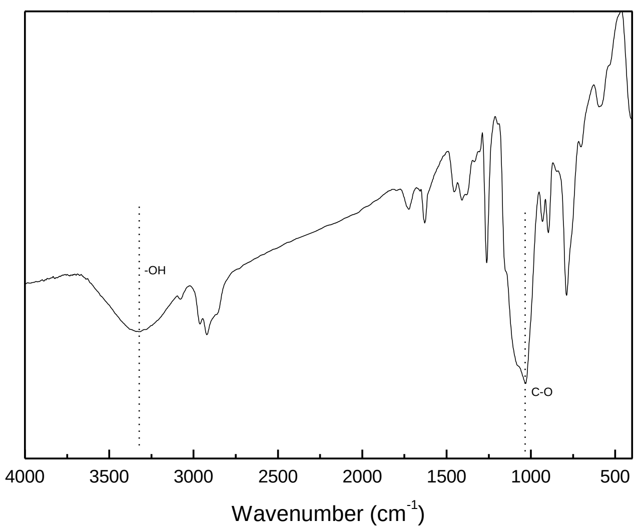

Figure S9. FTIR spectrum of **P3**

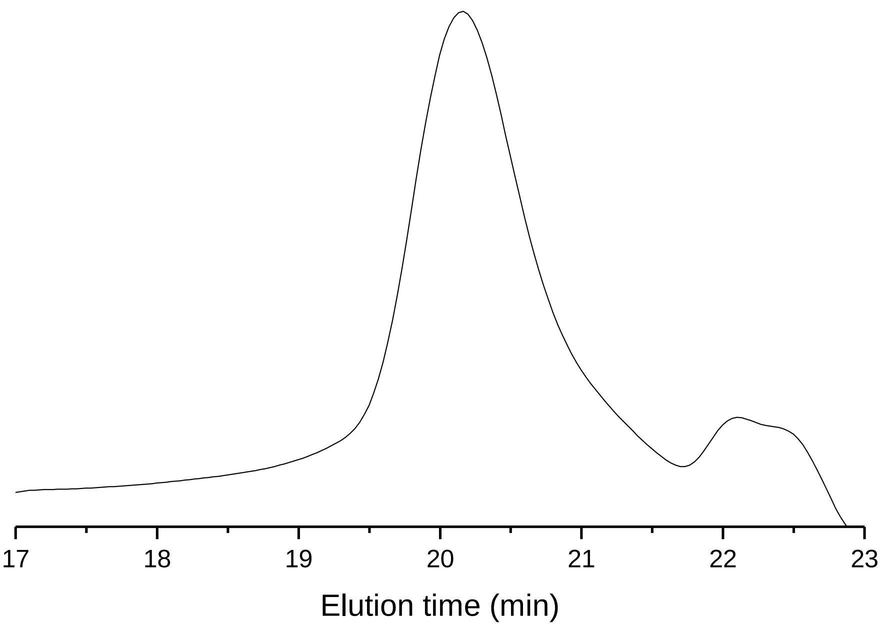

Figure S10. SEC elution cure of **P3**

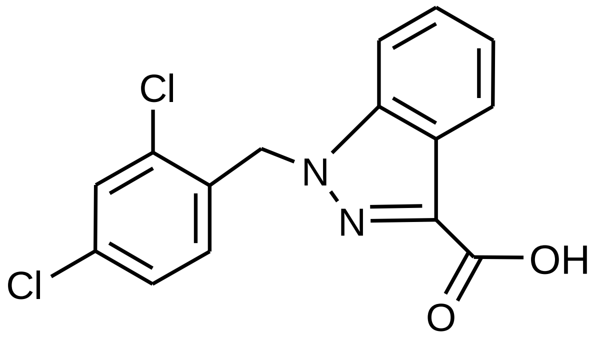

Scheme S4. Chemical structure of LND

5. References

(S1) Lai, J. T.; Filla, D.; Shea, R. *Macromolecules*, **2002**, *35*, 6754–6756.

(S2) Drohmann, C.; Moller, M.; Gorbatsevich, O. B.; Muzafarov, A. M. *J. Polym. Sci. Part A: Polym. Chem.,* **2000**, *38*, 741–751.

(S3) Tian, W.; Fan, X. D.; Liu, Y. Y.; Jiang, M.; Huang, Y.; Kong, J. *J. Polym. Sci. Part A: Polym. Chem.,* **2008**, *46*, 5036–5052.

(S4) Tian, W.; Lv, X. Y,; Huang, L. B.; Nazakat, A.; Kong, J. *Macromol. Chem. Phys.,* **2012**, *213*, 2450−2463.

(S5) Tian, W.; Lv, A. L.; Xie, Y. C.; Wei, X. Y.; Liu, B. W.; Lv, X. Y. *RSC Adv.,* **2012**, *2*, 11976–11987.

(S6) Aryal, S.; Prabaharan, M.; Pilla, S.; Gong, S. Q. *Int. J. Biol. Macromol.,* **2009**, *44,* 346–352.

(S7) Siepmann, J.; Peppas, N. A. *Adv. Drug Deliv. Rev.,* **2001**, *48*, 139–157.

(S8) Zuleger, S.; Lippold, B. C. *Int. J. Pharm.,* **2001**, *217*, 139–152.

(S9) Lemoine, D.; Francois, C.; Kedzierewicz, F.; Preat, W.; Hoffman, M.; Maincent, P. *Biomaterials,* **1996**, 17, 2191–2197.

(S10) Tian, W.; Fan, X. D.; Liu, Y. Y.; Jiang, M.; Huang, Y.; Kong, J. *J. Polym. Sci. Part A: Polym. Chem.,* **2008**, 46, 5036–5052.

(S11) Marcos, A. G.; Pusel, T. M.; Thomann, R.; Pakula, T.; Okrasa, L.; Geppert, S.; Gronski, W.; Frey, H. *Macromolecules*, **2006**, 39, 971–977.
